# Heat‐Mitigated Design and Lorentz Force‐Based Steering of an MRI‐Driven Microcatheter toward Minimally Invasive Surgery

**DOI:** 10.1002/advs.202105352

**Published:** 2022-02-03

**Authors:** Martin Francis Phelan, Mehmet Efe Tiryaki, Jelena Lazovic, Hunter Gilbert, Metin Sitti

**Affiliations:** ^1^ Physical Intelligence Department Max Planck Institute for Intelligent Systems 70569 Stuttgart Germany; ^2^ Department of Mechanical Engineering Carnegie Mellon University Pittsburgh PA 15213 USA; ^3^ Institute for Biomedical Engineering ETH Zurich Zurich 8092 Switzerland; ^4^ Department of Mechanical and Industrial Engineering Louisiana State University Baton Rouge LA 70803 USA; ^5^ College of Engineering and School of Medicine Koç University Istanbul 34450 Turkey

**Keywords:** active catheters, Lorentz force, magnetic resonance imaging, medical robotics

## Abstract

Catheters integrated with microcoils for electromagnetic steering under the high, uniform magnetic field within magnetic resonance (MR) scanners (3–7 Tesla) have enabled an alternative approach for active catheter operations. Achieving larger ranges of tip motion for Lorentz force‐based steering have previously been dependent on using high power coupled with active cooling, bulkier catheter designs, or introducing additional microcoil sets along the catheter. This work proposes an alternative approach using a heat‐mitigated design and actuation strategy for a magnetic resonance imaging (MRI)‐driven microcatheter. A quad‐configuration microcoil (QCM) design is introduced, allowing miniaturization of existing MRI‐driven, Lorentz force‐based catheters down to 1‐mm diameters with minimal power consumption (0.44 W). Heating concerns are experimentally validated using noninvasive MRI thermometry. The Cosserat model is implemented within an MR scanner and results demonstrate a desired tip range up to 110° with 4° error. The QCM is used to validate the proposed model and power‐optimized steering algorithm using an MRI‐compatible neurovascular phantom and ex vivo kidney tissue. The power‐optimized tip orientation controller conserves as much as 25% power regardless of the catheter's initial orientation. These results demonstrate the implementation of an MRI‐driven, electromagnetic catheter steering platform for minimally invasive surgical applications without the need for camera feedback or manual advancement via guidewires. The incorporation of such system in clinics using the proposed design and actuation strategy can further improve the safety and reliability of future MRI‐driven active catheter operations.

## Graphical abstract

Magnetic resonance imaging (MRI) systems have enabled the development of Lorentz force‐based tools such as active catheters for minimally invasive surgery. The world's smallest‐diameter, MRI‐guided, electromagnetic, active catheter with an outer diameter of 1 mm, is designed, modeled, and validated with 4° accuracy under a 7‐Tesla preclinical MRI.

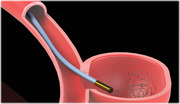

## 1 Introduction

Widespread use of noninvasive medical imaging modalities in surgeries such as magnetic resonance imaging (MRI), X‐ray, and ultrasound (US) has enabled the deployment of micron‐sized surgical tools such as guidewires and catheters into narrow cavities within the body. The conventional method for gaining access into a vessel, known as the Seldinger technique, involves gaining access to a blood vessel through the use of a puncturing needle, steerable guidewire through the needle, and preshaped or steerable catheter passed over the guidewire.^[^ ^1^ ^]^ However, various issues can still arise with this approach, such as vessel perforation, breakage of the device tip, kinking, looping, or loss of the guidewire.^[^ ^2^ ^]^ Guidewires and catheters can also be subject to losses in manual torque transmission to the distal tip in tortuous vessels, which makes navigation more challenging, especially when encountering narrow vessels located at small angles. Steerable (active) catheters and continuum robots offer an alternative to traditional passive guidewire‐based catheter deployment techniques with improvements in maneuverability and remote manipulation.^[^ ^3^, ^4^, ^5^, ^6^, ^7^ ^]^ The main challenge in active catheter design is transmitting force and torque through the soft slender body to actuate the catheter tip. Most commercial systems such as Sensei X and Magellan (Hansen Medical, Mountain View, CA, USA) use tendon‐based force transmission.^[^ ^8^, ^9^, ^10^ ^]^ However, researchers have proposed many different alternative actuation mechanisms, such as multi‐backbone,^[^ ^11^ ^]^ concentric tubes,^[^ ^12^, ^13^ ^]^ pneumatics,^[^ ^14^ ^]^ smart materials,^[^ ^15^, ^16^, ^17^, ^18^, ^19^, ^20^, ^21^ ^]^ hydraulics,^[^ ^22^, ^23^, ^24^, ^25^, ^26^, ^27^ ^]^ magnetics,^[^ ^28^, ^29^, ^30^, ^31^, ^32^, ^33^ ^]^ or hybrid approaches^[^ ^34^, ^35^ ^]^ to overcome certain drawbacks of tendon‐based systems.

**Figure 1:**
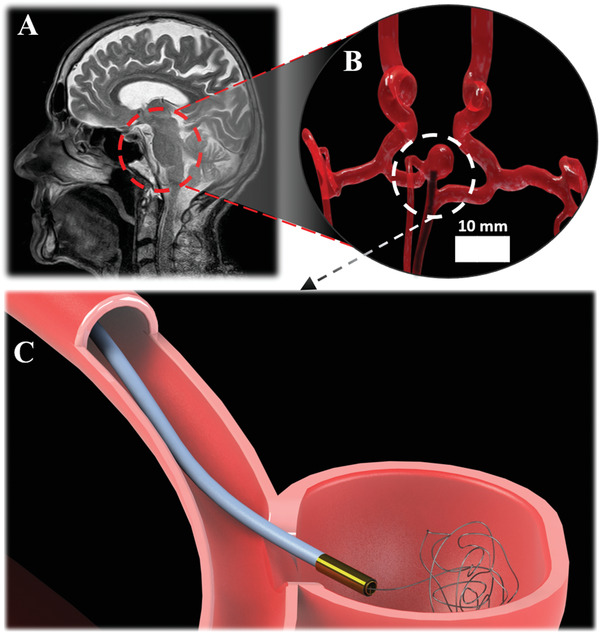
Schematic of an active, MRI‐driven, Lorentz force‐actuated catheter performing neuroembolization within the circle of Willis under MRI guidance. A) MR scan snapshot of head locating the circle of Willis rendered in B. B) Computer‐rendering of circle of Willis with active catheter (shown as shadowed outline due to image distortion) and aneurysm located by white boundary. C) Computer‐rendering of active catheter deploying embolization coil into aneurysm.

Integration to existing medical imaging modalities is another crucial part of device design. X‐ray imaging is currently the gold standard for real‐time visualization during minimally invasive surgeries. Radiopaque catheters can be visualized easily in vascular structures filled with contrast agents. However, it is hard to visualize the effect of the intervention on soft tissue with X‐ray imaging due to the poor soft‐tissue contrast. Therefore, there is a growing interest in MRI‐guided minimally invasive interventions due to the high soft‐tissue contrast of MRI images for visualizing blood vessels (such as within the brain; see **Figure** **1**) and tissue response as well as no ionizing radiation, real‐time tool tracking capabilities,^[^ ^36^, ^37^, ^38^, ^39^, ^40^ ^]^ and physiological measurement capabilities (e.g., MRI thermometry, diffusion, perfusion),^[^ ^41^ ^]^ However, MRI scanners impose new constraints on medical device design and actuation. First, the permanent high magnetic field limits material choices to nonmagnetic materials for device construction to avoid unintended magnetic force and torque. Second, large nonmagnetic metal objects cause imaging susceptibility artifacts. Last, the MRI scanner's radio‐frequency (RF) pulses can induce heating within conductive materials found in medical tools.^[^ ^42^ ^]^

Therefore, there are numerous studies on developing MR‐compatible actuation techniques for device steering.^[^ ^43^, ^44^ ^]^ These approaches include using smart materials,^[^ ^45^, ^46^, ^47^, ^48^, ^49^, ^50^ ^]^ hydraulic,^[^ ^51^, ^52^ ^]^ pneumatic,^[^ ^53^ ^]^ and MRI‐driven (magnetic) actuation.^[^ ^54^, ^55^ ^]^ Thermal actuation involves the use of current to induce forces and motion using thermally active materials that are highly responsive to changes in temperature, such as shape memory alloys (SMAs). SMAs can perform large deformations in small sizes for catheter steering. However, they generally require longer response times, demonstrate highly nonlinear behavior, and cause safety risks of heating neighboring tissue. Hydraulic actuation can transmit large forces through the slender catheter using fluid pressure. However, this fluid pressure over compliant continuum bodies causes radial expansion for positive pressures and buckling for negative pressures leading to stiffness variability and fatigue in the soft body. MRI‐driven actuation offers significant advantages over the aforementioned techniques due to its scalability, safety, response time (nearly instantaneous), accuracy (other methods experience nonlinearities in actuation), and degrees of freedom (DoF).^[^ ^56^ ^]^ In addition, MRI‐driven actuation can utilize imaging gradient coils user‐controlled to generate spatial field gradients for steering a wireless robot^[^ ^57^, ^58^, ^59^ ^]^ or magnetic catheter tip in 3D.^[^ ^60^, ^61^ ^]^ However, embedding magnetic elements to catheters for gradient steering introduces significant MR image distortion and additional catheter weight and bulkiness.

Another MRI‐driven actuation approach is mounting microcoils on the catheter tip for Lorentz force‐based catheter steering using manually‐wound^[^ ^62^, ^63^, ^64^ ^]^ or laser‐machined microcoils.^[^ ^65^ ^]^ Lorentz force‐based steering approaches introduce less weight to the soft body due to the high force‐to‐weight ratio in comparison to gradient steering.^[^ ^54^ ^]^ Moreover, the image distortion can be controlled since the image artifacts only occur when coils are activated. Magnetically‐assisted catheterization using microcoils has been shown to be faster than manual navigation using MR imaging guidance for larger angles and comparable to X‐ray guidance.^[^ ^66^ ^]^ However, microcoil‐based Joule heating effects have been a major design concern. Prior research has shown tissue thermal injury occurs above local temperatures of 44°C.^[^ ^67^ ^]^ Catheter‐integrated microcoil studies have indicated using currents inputs above 300 mA (1.2 W) can lead to vessel thrombus, vacuolization, and medial hemorrhage.^[^ ^68^ ^]^ Potential solutions have included integrating heat dissipation mechanisms, such as alumina to the catheter tip and passing saline coolant through the microcoil tip, or regulating current to less than 300 mA (1.2 W) with less than 1 min activation times.^[^ ^68^, ^69^ ^]^ However, such solutions introduce additional weight and bulkiness to the catheter tip, require flow through the catheter, limit working channel size, and constrain the time necessary for active steering in the workspace.

Here, we propose an alternative solution to the Lorentz force‐induced heating concern without using active cooling or limiting microcoil activation times for steering. This is accomplished using a heat‐mitigated design and actuation strategy using the previously mentioned 44°C as a heating threshold for safe navigation within the body (assuming no arterial flow; worst‐case scenario). Further improving upon existing microcoil design and manufacturing methods, a quad‐configuration microcoil design is introduced, allowing for more compact microcoil designs and tip weight reductions. This design enabled the development of the smallest, active, MRI‐driven quad‐configuration microcatheter (QCM) in the literature with a diameter of 1 mm for steering within confined MRI environments. Key performance parameters for MRI‐driven catheter designs were analyzed using a nonlinear continuum model for elastic bodies known as the Cosserat rod model. The Cosserat model allowed the formulation of a power‐optimized controller for maneuvering the microcatheter tip. It was also used to generate design lookup graphs for manufacturing other power‐optimized microcatheters according to insertion length and desired tip range of motion, depending upon the anatomical environment. Using the proposed design and actuation approach minimizes the tip stiffness and Joule heating effects to obtain large bending angles. We validated device safety using MRI thermometry to assess any tissue damage occurring within an ex vivo porcine kidney while keeping catheter performance goals in mind. Device tip steerability is tested in MRI‐guided feasibility experiments under a 7 T magnetic field using kidney tissue, workspace‐constrained narrow rings, and a neurovascular phantom model.

## 2 Results

### 2.1 Heat‐Mitigated Design and Lorentz Force Actuation

**Figure 2:**
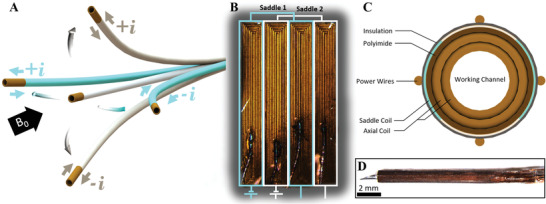
Images of the proposed active microcatheter design. A) Photo of the QCM microcoil design on a planar surface with circuit design for both saddle coil sets. B) Cross‐sectional view of the assembled active catheter. C) Photo of the manufactured microcoil assembled on a 25 G needle (Praxisdienst, Longuich, Germany) around the axial coil. D) Quad‐configuration extends the degrees of freedom (DoF) depending on current polarity and orientation with respect to the **B_0_** field.

Lorentz‐force actuators have proven to be effective for various robotic/medical applications due to their precision, high force output, and scalability for soft device integration.^[^ ^70^, ^71^, ^72^ ^]^ These actuators utilize external magnetic fields to generate a force directly controlled for robotic actuation. Therefore, Lorentz‐force actuators can utilize the high external magnetic field within MRI environments to develop robotic devices. Researchers have demonstrated integrating such actuators to catheters through the use of copper coils for Lorentz force‐based steering in blood vessels^[^ ^66^ ^]^ and the heart.^[^ ^73^ ^]^ In this approach, controlling microcoil current polarity directly translates to a tip deflection in the respective direction (**Figure** **2**A). The generated magnetic moment, **m**, and corresponding torque, **T**, can be determined in terms of the number of coil loops, *N*, current, *I*, and area normal vector, **A**, of a coil loop, and magnetic field vector within an MR scanner ($B_{0}=\left(0,0,B_{0}\right))$, where *B*_0_ is the uniform, permanent, magnetic field inside the MRI. This relation is represented as

(1) $$T=m\times B_{0}=NI\left(A\times B_{0}\right)$$

which can be used to govern axial coil torque for a Lorentz force‐actuated catheter with equally‐sized coil loops. In terms of saddle (side) coil implementation, microcoils can be integrated to the catheter tip for additional degrees of freedom (DoF). Liu et al. has shown manually‐wrapped copper wires facilitate manufacturing at the cost of tip weight and bulkiness, leading to larger catheter diameters on the order of 4–5 mm (see Figure S3A, Supporting Information).^[^ ^74^ ^]^ Design optimization of an MRI‐driven catheter using a four coil configuration allowed for maximizing the achievable workspace within the heart given certain constraints such as the number of coil sets and current inputs.^[^ ^75^ ^]^ However, maximizing the number of coil turns and area may not be feasible for navigating within narrow vasculature. On the other hand, laser lithography has been used to produce more compact designs by overlaying multiple coils down to 2 mm catheter diameters (Figure S3B, Supporting Information).^[^ ^65^ ^]^ In order to improve upon both existing design schemes, laser machining was used in conjunction with the Archimedian spiral coil design to create an in‐plane, quad‐configuration, microcoil design shown in Figure 2B,C and Figure S3C, Supporting Information. The proposed design enables more compact and significantly smaller final catheter diameters, down to 1 mm, than previously proposed designs while achieving comparable steerability (see Figure S1, Supporting Information). In this approach, both saddle coil sets are integrated on the same circumferential plane without introducing additional layer thickness compared to literature. The governing equations for a rectangular Archimedian spiral are given below for estimating the microcoil's magnetic moment. The approximate effective area of all coil loops can be expressed as

(2) $$A_{\mathrm{total}}=\sum_{i=0}^{N-1}\left(L_{c}-w-i\left(w+2t\right)\right)\left(W_{c}-w-i\left(w+2t\right)\right)$$

and corresponding total wire length for estimating power consumption as

(3) $$L_{\mathrm{coil}}=\sum_{i=0}^{N-1}2\left(W_{c}+L_{c}-2i\left(2t+w\right)-2w\right)$$

Inserting Equation (2) into Equation (1) yields the magnetic moment of a single saddle coil

(4) $$m=IA_{\mathrm{total}}$$

Achieving higher bending angles for Lorentz force‐based actuation implies maximizing the coil's magnetic moment. As shown in the above equations, tuning various parameters (i.e., coil turn number, current, catheter diameter) can influence the performance. Typically, a larger coil turn number implies better bending performance due to the increasing coil area. However, when power is constrained to the heating threshold (0.5 W) to mitigate heating effects, an inverse relation exists between the magnetic moment and coil turn number (see Figure S2, Supporting Information). In other words, a lower coil turn number is ideal for mitigating heat but requires higher currents to generate such magnitudes of electromagnetic torque leading to undesirable heating within the power wires. However, using larger power wires to mitigate heating increases flexural rigidity depending upon wire gauge and thus has undesirable effects on catheter steerability. Therefore, for this study, a microcoil turn number of 7 is used to maximize the magnetic moment while remaining within well‐established current ratings for power wires and an acceptable range of stiffness for endovascular catheters. However, Joule heating remains to be a concern; therefore, optimizing microcoil (saddle/axial) power distribution is imperative to catheter performance.

### 2.2 Nonlinear Quasistatic Continuum Model

Steerable catheters undergo large deformations/motions during surgical procedures. One method of modeling such motions commonly used to model elastic rods and continuum rods is the Cosserat rod theory.^[^ ^76^, ^77^, ^78^, ^79^ ^]^ The Cosserat rod model integrates the traditional bending and twisting of Kirchhoff rods with additional stretching and shearing to capture full beam dynamics. The Cosserat model accurately depicts the nonlinear dynamics of elastic rods with different materials and geometries.

For the purposes of this study, the catheter is modeled as a cantilever beam undergoing an external torque and tip force. We describe the state of the catheter using a set of *N* discretized segments $Y={\left[y_{0}^{T},y_{1}^{T},\text{…},y_{N}^{T},\right]}_{N}^{T}$. The discretized state vector for each segment *i* contains segment position ($p_{i}\in R^{3}$), orientation ($R_{i}\in SO\left(3\right)$), extension force ($n\in R^{3}$), and shear torque ($m\in R^{3}$), which can be expressed in one vector $y_{i}:=\left[p_{i},R_{i},n_{i},m_{i}\right]$. The rotation matrix is defined in the MRI's fixed coordinate frame I, along with two additional coordinate frames; control frame C representing the catheter free length starting position and tip frame **T** locating the start of the microcoils (see Figure S4, Supporting Information). Therefore, a system of nonlinear ordinary differential equations (ODEs) can be expressed as

(5) $$\begin{array}{cc} {\dot{p}}_{i} & =R_{i}v_{i} \end{array}$$

(6) $$\begin{array}{cc} {\dot{R}}_{i} & =R_{i}u_{i} \end{array}$$

(7) $$\begin{array}{cc} {\dot{n}}_{i} & =-\rho Ag \end{array}$$

(8) $$\begin{array}{cc} {\dot{m}}_{i} & ={-\dot{p}}_{i}\times n_{i} \end{array}$$

where v and u are tangent and curvature vectors defined as, $v=\hat{z}+K_{1}R^{T}n$ and $u=K_{2}R^{T}m$, where $\hat{z_{i}}$ is the unit vector in local coordinate frame, **K**_***1***_ = diag(*GA*, *GA*, *EA*) and **K**_2_ = diag(*EI*_A_, *EI*_A_, *GJ*). *G*, *A*, *E*, *I*_A_, and *J* represent the shear modulus, cross‐sectional area, elastic modulus, area moment of inertia, and polar moment of inertia, respectively.

Catheter forward kinematics, $Y=f(n_{0},m_{0})$, can be calculated through numerical integration using fourth order Runge–Kutta algorithm, given the catheter's initial conditions: $R_{0}=R_{C}$, $p_{0}=p_{C}$, $n_{0}=n_{C}$, and $m_{0}=m_{C}$. Although the forward kinematic model in an initial value problem form is useful for simulating catheter motion given a base wrench, an inverse kinematic model is needed to determine the minimum catheter torque for reaching desired orientations.

In this study, an inverse kinematic model in a boundary value problem (BVP) form is formulated with the following boundary conditions: $R_{0}=R_{C}$, $p_{0}=p_{C}$, $n_{T}=0$, $m_{T}=\tau_{\text{des}}$ and $R_{T}=R_{\text{des}}$. Here, we express $n_{T}$ and $m_{T}$ as the magnetic wrench at the tip of the catheter. Due to the negligible magnetic gradient pulling force acting on the catheter tip in comparison to the magnitude of a distributed Lorentz force, we assume there is only torque at the tip. Therefore, the inverse kinematic for desired tip torque ($\tau_{\text{des}}=\text{IK}\left(R_{T,\text{des}}\right)$) is calculated by solving the following optimization problem for tip torque

(9) $$\begin{array}{cc} \underset{Y}{\mathrm{arg min}} & \left|\right|R_{T,\text{des}}⊟R_{T}{\left|\right|}_{2}^{2}+\left|\right|\tau_{\text{des}}{\left|\right|}_{2}^{2}\\ \text{s.t.}\; & Y=f(n_{0},m_{0}) \end{array}$$

where the box minus ($⊟:\text{SO}\left(3\right)\times \text{SO}\left(3\right)\to R^{3}$) is the rotation difference operator based on the matrix logarithm defined in Lie algebra.^[^ ^80^ ^]^ Tip torque is $\tau_{\text{des}}=m_{N}$. Optimization is solved in real‐time using the iterative Levenberg–Marquardt method implemented in C++,^[^ ^78^ ^]^ where catheter forward kinematics is used as the shooting function. It is important to note that the ⊟ error is essential for the stability of the solution for near singular values, and the quadratic on tip torque regularizes the cost function to eliminate inverse kinematic solutions with loops.

### 2.3 Power‐Optimized Coil Current Current Distribution and Control

**Figure 3:**
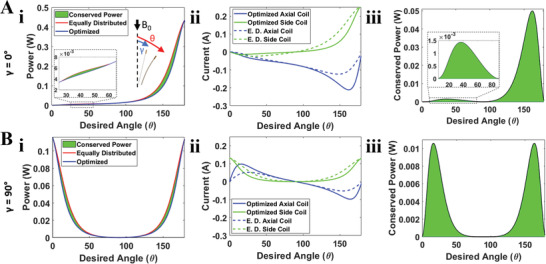
Power‐optimized controller capabilities at various initial orientations and their corresponding the Joule heating effects when coils are powered. A‐i) defines the initial angle between the catheter tip and **B**_**0**_ field vector, γ, and final tip orientation, θ, upon excitation. A,B) represent when the QCM begins aligned with and perpendicular to the **B_0_** field, respectively. Power was compared between both actuation schemes in (A‐i) and (B‐i): equally‐distributed (E.D.) power and optimized current distribution between coils. Conserved power using optimal approach indicated in green. (A‐ii) and (B‐ii) show the current distribution using both actuation schemes. (A‐iii) and (B‐iii) show total power conserved using the power‐optimized controller.

Microcoil‐based heat generation can be reduced by optimally distributing current to the side and axial coils. The tip orientation controller is therefore comprised of a two‐stage optimization scheme: 1) inverse kinematics to determine torque using Equation (9), and 2) saddle/axial coil current distribution. A power‐optimized current distribution problem is formulated as a nonlinear quadratic optimization

(10) $$\begin{array}{cc} I^{*}=\underset{I}{\mathrm{arg min}} & \left|\right|\tau_{\text{coils}}-\tau_{\text{des}}{\left|\right|}^{2}+{\alpha ||I||}_{R}^{2} \end{array}$$

(11) $$\begin{array}{cc} \text{s.t.}\; & \tau_{\text{coils}}=\tau_{\text{side},1}+\tau_{\text{side},2}+\tau_{\text{axial}} \end{array}$$

where $I=[I_{\text{side},1},I_{\text{side},2},I_{\text{axial}}]$ represents the saddle and axial coil currents, and τ_coils_ represents the total torque generated by a saddle and axial coil set, respectively. The first term of the cost function is for consistency between desired tip torque and total coil torque, and second term is the coil power consumption cost, where $R=\text{diag}(R_{\text{side},1},R_{\text{side},2},R_{\text{axial}})$ is the resistance of the coils. Due to the difference in magnitude between torque error and induced currents, an α constant was incorporated (determined using a grid search to find the best fitting; 1 × 10^−6^). This optimization is also solved using the Levenberg–Marquardt method. A comparison between actuating coils using equally‐distributed power versus the optimal approach is shown in **Figure** **3**.

The current distribution optimization minimizes power consumption and Joule heating effects by prioritizing the axial coil when achieving angles between 90° and 160° (Figure 3A‐ii), resulting in an increase in the conserved power. Beyond 160°, the side coil generates more torque resulting in current reprioritization and thus a loss in the conserved power. This phenomenon is also observed when γ = 90° (Figure 3B‐ii); between 20° and 50°, the axial coil is prioritized. Angles less than 20° cause current redistribution toward the side coil as the catheter aligns parallel to the **B**_**0**_ field. Due to the differences in coil resistance between the saddle and axial coil, significant power changes can be observed (Figure 3A‐iii,B‐iii). Figure 3A‐iii demonstrates 3 mW of conserved power for smaller angles and as much as 50 mW for larger angles when the catheter begins aligned with the **B**_**0**_ field. Figure 3B‐iii indicates a conserved power magnitude of 10 mW when γ = 90° and the catheter aligns parallel to the **B**_**0**_ field. Figure S5, Supporting Information demonstrates as much as 25% conserved power regardless of initial orientation. Such power conservation improves overall catheter safety during steering at low rotation angles, and increases the catheter workspace up to 10° at higher angles.

However, the obtained optimized current values are only valid for a desired tip orientation for the quasi‐static case. Applying calculated currents from an arbitrary initial orientation does not guarantee the QCM to reach the desired orientation, especially if the catheter tip rotates through singularity points running parallel/perpendicular to **B**_**0**_. Moreover, large tip rotations can cause overshoots. Both of these issues can be overcome by incrementally controlling the coil currents. This quasi‐static open‐loop control scheme could be combined with path planning to be dynamically optimal in future work.

### 2.4 Model‐Based Scaling Analysis for Constrained Insertion Lengths

To maximize bending performance for fixed catheter lengths in confined workspaces such as the kidney or heart, multiple design variables such as coil length, power wire size, and catheter diameter need to be considered. It is not fully understood to what degree each of these parameters impacts overall catheter safety and bending. For instance, an optimal microcoil/catheter insertion length ratio under safety constraints for active, MRI‐driven catheters has not yet been determined because minimizing the coil length can reduce tip rigidity but at the cost of magnetic moment and additional power consumption.

Therefore, the Buckingham π theorem was applied to the given problem to create a set of independent nondimensional design parameters for optimal design scaling analysis. The nominal values for this analysis were based on commercial microcatheter design specifications such as insertion length (6 cm) and commonly used catheter materials (polyurethane). Ranges for catheter diameter (*D*_C_), coil length (*L*_C_), and power diameter (*pw*_D_) were as follows: *D*_C_ = [0.5, 4] mm; *L*_C_ = [0.5, 15] mm; *pw*_D_ = [50, 150] µm. Performance was measured using a goal of 180° of bending using minimal power/heating. Saddle coil wire thickness, *t*, was based on availability of raw materials.

Given the design variables in this study, seven π groups were found (12)

(12) $$\theta =f\left(\frac{L_{c}}{L},\frac{W_{c}}{L},\frac{EL}{B_{0}I},\frac{I_{A}}{L^{4}},N,\gamma \right)$$

**Figure 4:**
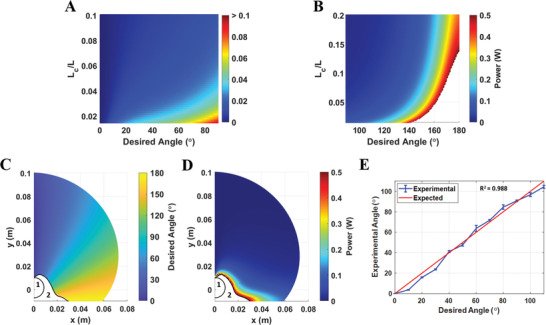
Heat map lookup graphs for various coil/catheter lengths in terms of power consumption to achieve desired tip orientations for A) small angles θ < 90° and B) large angles θ > 90° (area in white represents power requirements higher than the 0.5 W heating threshold). C) Desired tip orientation with respect to **B**_**0**_ field across workspace. D) Power consumption for QCM with various insertion lengths up to 10 cm. The QCM was assumed to be fixed at (*x*, *y*) = (0, 0). Area denoted by 1 and 2 represent the unachievable workspace due to coil rigidity and high power requirements, respectively. E) Linear correlation between the expected Cosserat model and experimental results.

Results for scaling laws governing the microcoil/catheter length, power consumption, and tip orientation show power wire diameter has the highest overall impact on catheter performance, in which minimizing the wire lead diameter reduces power consumption. Additionally, minimizing the catheter diameter and coil length also contributed to lower power consumption. Therefore, for the final design, certain design constraints were imposed based on scaling analysis and to facilitate manufacturing. The lumen was constrained to fit a 500 µm laser through the inner channel in order to minimize catheter diameter while also integrating tools to simulate minimally invasive surgery. Coil/catheter length ratios were examined closely to determine the optimal ratio for desired tip orientations. **Figure** **4**A indicates no more than 100 mW is needed to achieve angles less than 100° for any given coil/catheter length ratio. Figure 4B indicates higher heat generation (>0.5 W) to achieve angles above 150° for certain coil/catheter length ratios, indicated by the area in white. This heat generation for achieving larger angles at smaller ratios shows the relation between reducing microcoil magnetic torque output and stiffness at an attempt to improve the workspace. Figure 4A,B thereby form a design lookup graph for determining the best fitting coil/catheter insertion length ratio to safely achieve a specific tip orientation for a given application.

A microcoil/catheter length ratio of 0.16 and power wire diameter of 80 µm were used to reduce the tip weight/stiffness and facilitate manufacturing. With the introduction of six power wires to power all coil sets, the resultant composite modulus was determined to be 71.74 MPa, comparable to commercially available microcatheters. MRI **B**_**0**_ field strength was set to 7 T to coincide with our in‐house MR scanner. One tri‐coil (saddle/axial) bundle was integrated to the tip to demonstrate its steering and safety performance while minimizing catheter weight/stiffness. Final design parameters for the optimized QCM used in feasibility experiments are shown in Table S1, Supporting Information.

Last, we simulated the workspace of the proposed QCM using a power‐optimized controller. The tip orientation angle in the horizontal plane and required corresponding powers are shown in Figure 4C,D. The minimum insertion length was set to the coil length (1 cm), therefore there is an unachievable 1 cm radius workspace (denoted by 1) around the fixed point located at (*x*,*y*) = (0,0) due to the coil rigidity. However, it can be seen that large deformations up to 180° are possible for catheter lengths ranging up to 10 cm using less than 0.5 W. Moreover, areas in white, denoted by 2, are also infeasible due to high power requirements. Later, we compared the simulated tip orientation with the experimental data using a 6 cm catheter (Figure 4E); there is a linear correlation between desired and experimental angles with a root‐mean‐square error of 4°. We limited the workspace characterization to the horizontal plane since most of the catheter steering in practical cases occurs in the near horizontal plane. However, Figure 4C,D could be used to approximate tip angle and required power for motion in *Z* direction for small tip angles due to the axial symmetry and small microcoil mass. On the other hand, large tip deflections in *Z* direction would be affected by gravity severely. The catheter would only be stable when the catheter is inserted in a downward orientation as demonstrated by Liu et al.^[^ ^81^ ^]^ for unconstrained motions. Stable 90° deflection in the *Z* direction cannot be achieved by a single quad microcoil design due to actuation singularities; however, multiple quad coil sets integrated to the catheter^[^ ^81^ ^]^ could achieve stable upward motion in the future.

### 2.5 Endovascular Steering through Narrow Rings

**Figure 5:**
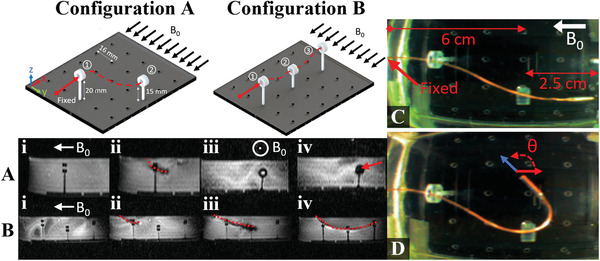
Experimental setup used to demonstrate the QCM steering capabilities in realistic, confined workspaces. A,B) Schematic of the confined ring experiments demonstrating the QCM DoF and controlled steering under MRI‐guidance. Red centerlines shown in (A‐i) show the QCM centerline passing through rings. (A‐iii,iv) show MRI‐guidance from the coronal plane assisting the QCM in steering the tip through the second ring, indicated by red arrow. B) MR image snapshots of the QCM navigating through three rings at different heights along the sagittal plane. C,D) Camera images demonstrating the QCM workspace capability, orienting its tip at 150° with respect to the **B**_**0**_ indicated by θ at a 9.5 cm length.

The QCM was teleoperated using joystick control through anatomically constrained rings with a 4 mm inner diameter submerged in water to simulate endovascular blood vessel navigation (see Movie S1, Supporting Information). Power was constrained according to model‐based inputs and monitored over time. Rings were set on a grid pattern with holes equally spaced at 16 mm to replicate confined workspaces such as the kidney.^[^ ^82^ ^]^ Configuration A demonstrates QCM dexterity gained from the tri‐coil design to achieve various desired tip orientations in different planes/image slices. The catheter was fixed 6 cm away from the second ring with an initial elevated height to prevent water leakage (**Figure** **5**A). MRI‐guidance from the side, or sagittal, view shown in Figure 5A‐i,ii shows the microcatheter entered through the first ring indicated by the red centerline. Navigation through the second ring was achieved using a front, or coronal, view on a repositioned image slice using real‐time projection imaging (Figure 5A‐iii,iv). The QCM tip can be tracked using the current‐induced microcoil image distortion as indicated by the red arrow in Figure 5A‐iv. In configuration B, the QCM is shown maneuvering through the image slice at different elevations Figure 5B. The vertical travel distance between rings is 5 mm as depicted in Figure 5A. In Figure 5B.i–iii, the QCM gained entry through the first and second ring using both axial and side coil sets (Figure 5B‐iii). However, in Figure 5B‐iv, the QCM consumed more power to steer against its pinned body and own weight to reach the third ring. However, total power consumption for all experiments never exceeded 50 mW. Due to the confined workspace in the MRI and limited camera field‐of‐view, demonstrating angles larger than 90° at γ = 0° is challenging. However, Figure 5C,D shows catheter capabilities under the power threshold, achieving a tip orientation of 150° using a ring as a pinned joint.

### 2.6 Ex Vivo Kidney Collecting System Navigation toward Laser Lithotripsy

**Figure 6:**
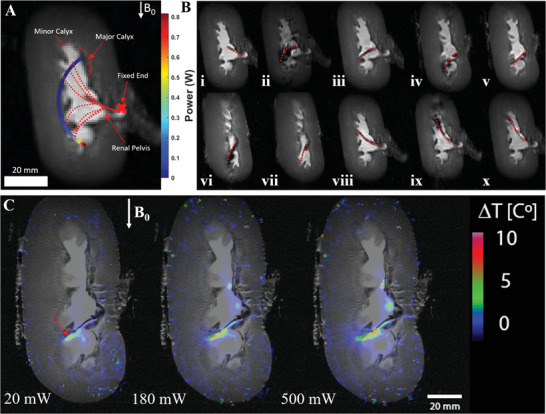
A) Ex vivo MRI thermometry‐based temperature maps for the QCM microcoil heating at power inputs of 20, 180, and 500 mW. Localized catheter tip heating is indicated by the red arrow in the first image. B) Simulated QCM workspace within the kidney (color‐mapping) MR image and experimental QCM trajectories (red dotted lines) for steering toward minor calyxes. Tip orientations and positions are color‐mapped according to consumed power from initial orientation with respect to the **B**_**0**_ (γ = 90°). B) Kidney steering in a confined workspace using variable QCM insertion lengths indicated in red. (B‐i–iii) Middle minor calyx navigation where the QCM reaches target position and holds orientation when power is off. (B‐iv–vii) Kidney navigation toward lower calyxes at higher power thresholds due to desired tip orientations/initial orientation. (B‐viii–x) Same workspace routine mirrored for upper minor calyxes.

The feasibility of navigation was tested using a porcine kidney tissue filled with Ringer fluid. The QCM was inserted in the renal cavity of the kidney through the ureter, mimicking a ureterorenoscopic surgery. Later, the QCM was navigated in calyxes to reach the collecting system through the renal cavity. Using power‐constrained thresholds from MRI thermometry and model‐based inputs, it was observed that the QCM can safely achieve the workspace of the kidney using minimal power using predominately axial coil excitation (**Figure** **6**A). The theoretical model was tested by fixing the QCM at a free length of 4 cm at the renal pelvis. The QCM is shown navigating the confined kidney workspace to simulate a kidney stone operation by first navigating the middle minor calyxes using power thresholds no more than 80 mW (Figures 6A‐i–iii). The QCM was repeatedly linearly actuated to reach other areas of the kidney while avoiding buckling. The QCM was advanced and steered deep within lower calyxes to reach a stone (Figures 6A‐iv–vii). Navigating these deeper regions required either rotation of the QCM or an increase in power from 80 to 500 mW. The procedure was completed by retracting the QCM 2 cm and steering back to the starting position in the middle major calyx to repeat a similar routine in the upper minor calyxes (Figures 6A‐viii–x) and (see Movie S2, Supporting Information).

### 2.7 Neurovascular Navigation toward Neuroembolization

**Figure 7:**
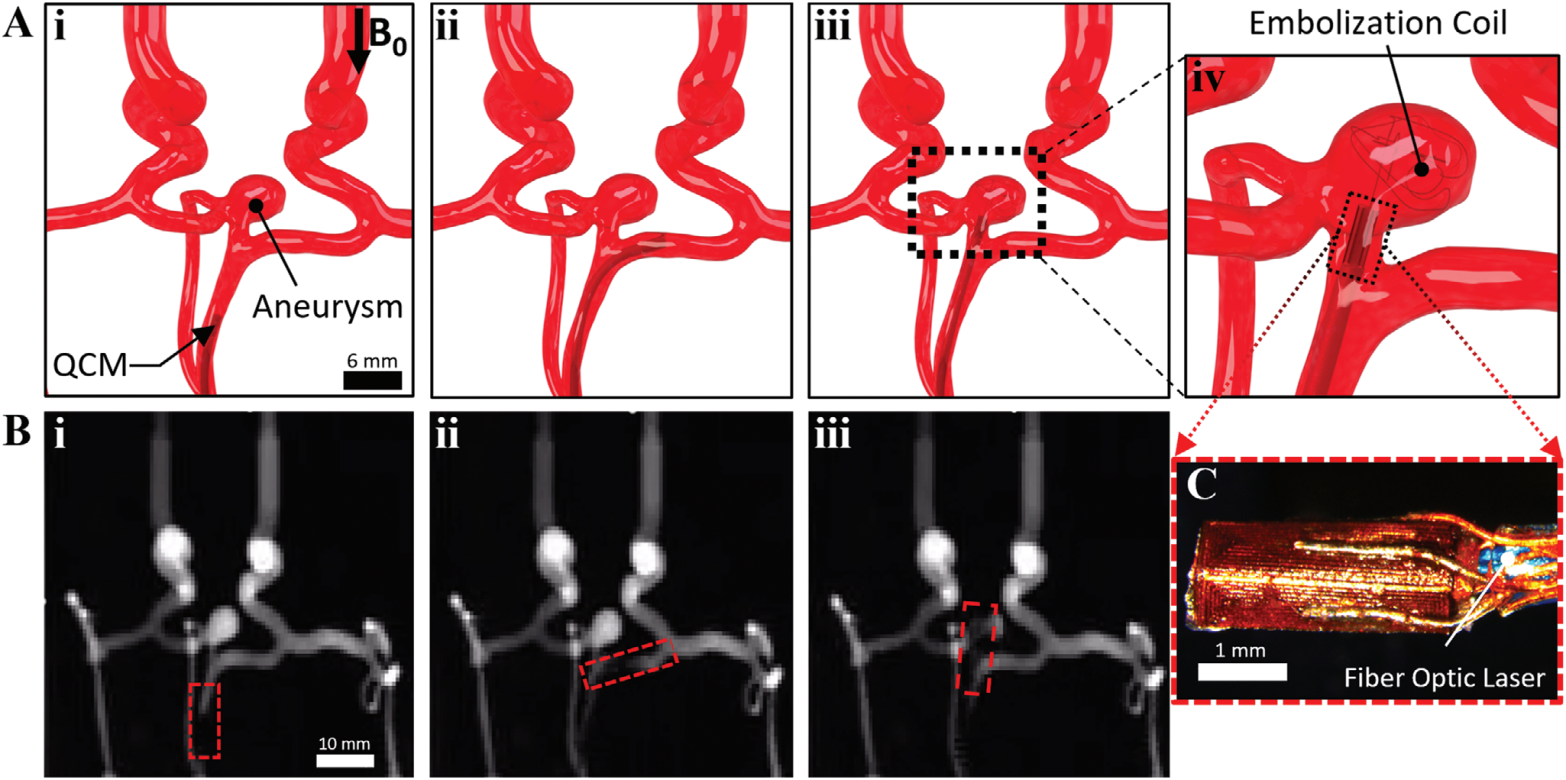
A) The circle of Willis 3D‐computer rendered model and starting position for 3 mm coil length QCM and neuroaneurysm (A.i). A‐ii) QCM taking a right turn at low power. A‐iii) QCM deploying embolization coil to treat aneurysm (magnified depiction in (A‐iv). B) Entire procedure replicated experimentally using MRI‐guidance, where the QCM is tracked by image distortion during Lorentz actuation indicated by the red box. C) Photograph of the QCM with an integrated fiber optic laser.

Due to the miniaturization of existing active MRI‐driven microcatheters, the QCM has gained the capability for neurovascular navigation through cerebral arteries, such as the circle of Willis which is located within the inferior side of the brain. A phantom silicone model of the circle of Willis (M3DP UG, Magdeburg, Germany) was used to demonstrate QCM steering under MRI guidance (**Figure** **7**A). The QCM was navigated upward through a <2 mm channel vessel indicated by the red box toward the cerebral aneurysm (Figure 7B‐i), steered to the right (Figure 7B‐ii) to demonstrate maneuverability, and navigated to the aneurysm to deploy an embolization coil (Figure 7B‐iii) and (see Movie S3, Supporting Information). Figure 7C shows a photograph of the QCM used for this study with a coil length of 3 mm along with the introduction of a fiber optic laser. Further details and photos in regards to the embolization coil deployment process are shown in Figure S7, Supporting Information, in which the embolization coil is deployed outside of the MRI scanner as a proof‐of‐concept.

## 3 Discussion

This work has shown the design and actuation of an MRI‐driven, active microcatheter for minimally invasive procedures. A novel quad‐configuration microcoil design was introduced to extend QCM DoF without significant weight/bulkiness to the catheter tip. In addition, the Cosserat model was implemented for the first time in an MR environment to demonstrate and provide an optimized design and actuation strategy for safer catheter steering. The model was validated with a root‐mean‐square error of 4°, given unknowns in the experiment such as random wire tensioning within the lumen due to fixturing, microcoil mounting misalignment on the catheter tip/misalignment during testing, and a confined workspace. However, due to the confined workspace of blood vessels and angles needed for navigation (20–60°), validation experiments were sufficient to demonstrate the validity of the Cosserat model for MRI‐driven, active catheter steering.

The Cosserat model was utilized in scaling analysis to inform the design process for an optimal, MRI‐driven catheter. Maximizing magnetic torque on the tip is highly dependent on power and coil length; therefore, a design lookup graph was presented as a heat map demonstrating minimal power generated to achieve necessary angles at corresponding coil length/catheter length ratios.

Using the power‐optimized controller, the magnitude of conserved power depends upon the catheter's initial orientation and desired tip orientation. However, regardless of initial orientation, one can expect as much as 25% conserved power for angles near parallel to the **B**_**0**_ when compared to equally distributing currents. Such conserved power can then be used for achieving higher angles to increase the catheter range of motion. It is concluded that higher power is conserved when the axial coil can be prioritized over the saddle coil for a given desired orientation. However, conserved power is minimized as the catheter aligns with the **B**_**0**_ field. Therefore, results indicate that an MRI‐driven catheter's initial orientation should be considered when planning a procedure to reduce tissue heating effects. However, the proposed power management controller substantially improves heat mitigation regardless of the catheter's initial orientation.

Precision in the microcoil machining process is key to fabricating QCM prototypes. Laser cutting parameters such as mark speed and laser power were critical in the coil machining step alone and highly dependent on substrate preparation. Therefore, lithography parameters should be carefully tuned to a specific coil design. However, using the novel design, minimal tip bulkiness/weight from one coil set was added to the catheter tip, allowing for the design of microcoil arrays along the microcatheter shaft for additional DoF.

All feasibility experiments demonstrated maneuverability without surpassing the maximum power threshold. Additionally, experiments were conducted under a worst‐case condition with no arterial flow which would further aid in heat dissipation. QCM dexterity was shown using vessel rings fixed at different planes and locations using a teleoperated, remote‐controlled catheter steering system. The QCM was able to achieve desired tip orientations using minimal power consumption for navigating the confined space between rings. The flexible length of the catheter is also challenging to determine solely off MRI‐guidance, therefore real‐time catheter tracking as demonstrated in other studies^[^ ^38^ ^]^ should be incorporated in future work.

Kidney steering was shown to be feasible for lower and upper calyxes with a realistic 90° initial orientation using low power consumption (<50 mW). Due to high friction between the QCM and tissue, higher power thresholds (<500 mW) were applied instantaneously to remove the catheter from pinned joints. These results demonstrate the potential use case for kidney stone laser lithotripsy, where steerable catheters are needed for breaking up large kidney stones. However, achieving higher angles at the same initial orientation may be unsafe depending upon the workspace and the desired tip orientation. This should be considered when planning safe remote‐controlled MRI‐guided catheter operations. Another medical application for the QCM could include cardiac ablation, where steerable catheters have proven to be useful in correcting irregular heart signaling pathways. With the current approach, the QCM would improve upon previous proposed devices using lower power consumption to achieve similar tasks. Neurovascular steering demonstrated manufacturing scalability and QCM steering capability. Low power thresholds were more than sufficient to replicate a neuroembolization procedure. Future work can focus on scaling the device down further to achieve smaller vasculature as well as reducing tip stiffness to reduce chances of blood vessel blunt force trauma. Additionally, commercialization of 7 T MR scanners (SIGNA 7T, GE Healthcare, Chicago, Illinois) for clinical use has promoted improvements in active, MRI‐driven, electromagnetic catheter designs for safer navigation within confined workspaces commonly found in neurological and urological interventions.

## 4 Conclusion

Robotically actuated catheters may offer many advantages to the surgical operations including increased surgical precision and improved patient outcomes. These devices can be tracked and actuated using the MR scanner's strong, external, magnetic field, and individually controlled microcoils to steer in confined environments such as the kidney or cereberal arteries. In this paper, we introduced a quad‐configuration microcoil design and optimization strategy for scaling down future MRI‐driven catheter designs. This work has further explored and addressed potential heating concerns by presenting a power‐optimized steering strategy for confined workspaces. Future work will focus on integrating additional coil sets for increased dexterity and using the validated Cosserat model for MRI‐based catheter shape tracking and motion planning for endovascular navigation. These advancements will further prove the viability of MRI‐driven, active catheters for their future clinical use.

## 5 Experimental Section

### QCM Fabrication and Assembly

Previous work on laser lithography‐based microcoils has shown success using a custom cylindrical lithography system.^[^ ^66^ ^]^ This paper uses an alternative method to fabricate microcoils using a conventional flexible circuit fabrication approach for manually‐wrapped microcoils. This approach ensured uniformity of the copper layer and highly precise circuit patterns. Microcoils can be machined down to the resolution of the laser cutting machine (Protolaser U3, LPKF) and were only limited to the circumferential surface area of the medical device.

The process begins by manufacturing the axial coil forming the rigid core. The coil was manually‐wound on a 25 G needle to the desired coil length and coated using a transparent silicone spray (494–714, RS Components, Frankfurt, Germany). After 2 h to cure the silicone, a second coil layer was wrapped around the axial coil and sprayed for additional insulation. The quad coil was fabricated by gluing an 18‐µm thick annealed copper sheet with a polyimide backing layer (Goodfellow, Hamburg, Germany) onto a glass slide using cyanoacrylate with little to no air entrapment. Parametric planar microcoil designs were generated in SolidWorks and laser‐cut using optimized cutting parameters to produce high precision copper microcoils. Each microcoil was released from glass by immersion in a warm water. Remaining cyanoacrylate backing the microcoil was dissolved using acetone in a glass beaker. Microcoils were soldered using copper magnet wire with a microsoldering station and insulated with a 20 µm thick parylene layer. After insulation, they were manually‐wrapped around the axial coil and secured in place with cyanoacrylate (see Figure S1A, Supporting Information). The resulting coil bundle was attached to a 3 Fr Pellethane thermoplastic polyurethane microcatheter (Nordson Medical, Salem, NH) (see Figure S1B). Wire leads were passed directly through the microcatheter lumen to minimize bending stiffness and current‐induced image distortion surrounding the catheter. Copper wires 400 µm in diameter were soldered to the wire leads for connection to a terminal block. The final prototype was a 3‐DoF tri‐coil microcatheter with a final outer diameter of ≈1 mm (Figure 2D).

### Coil Characterization

Microcoil laser‐cutting quality was measured using each saddle coil's resistance as the machining metric. The theoretical resistance was calculated using Equation (3) and material manufacturer specifications. Microcoil machining repeatability was validated with 50 laser‐cut microcoils. The error was 3% from the expected value (6.22 Ω), consistent with laser‐cutting machine specifications, thereby validating the microcoil machining process was both reliable and effective for development of QCM prototypes.

### Experimental Setup

The QCM was controlled using two dual motor drivers (DRV8835, Pololu) for microcoil actuation and a nonmagnetic piezoelectric motor (LS15, Piezomotor) for linear actuation (see Figure S6, Supporting Information). The microcatheter controller was implemented using a custom C++ interface. The microcatheter was fixed on a 3D‐printed platform mounted to a linearly sliding, manual bed of a 7 Tesla preclinical MRI (BioSpec 70/30 USR, Bruker, Ettlingen, Germany), actively shielded gradient (BGA20SHP) system with a maximum 300 mT m^−1^ gradient strength and 154 mm quadrature birdcage coil. Power cables for the QCM extended from the MRI into the adjacent computer room to limit equipment in the strong, external, magnetic field. The inside bore was configured with a custom camera setup consisting of one MR‐compatible camera (12M, MRC) for monitoring QCM motion from the top plane.

### Single Direction Work‐Space and Orientation Dependence

To explore the workspace, model‐based inputs determined using the power‐optimized algorithm were used to validate the nonlinear model. The experimental environment consisted of the QCM cantilevered with a fixed proximal end and a 6 cm free‐length suspended in a stagnant, room temperature, water bath. Due to the confined workspace, experimental angles were tested up to 110° at 10° increments. Corresponding inputs for saddle and axial coils were set accordingly in three separate trials, allowing the microcatheter to achieve its resting orientation after each excitation. The QCM's initial orientation was adjusted after each trial to ensure a colinear alignment with the **B**_**0**_ field. Angles were captured from the top plane and post‐processed using ImageJ and grid paper as a reference. The nonlinear model was used to simulate the QCM's workspace for different insertion lengths, tip orientations, and power inputs (Figure 4C,D) as well as the initial orientation, γ, dependence's effect on angle and power requirements for when γ = 0° (Figure 3A) and γ = 90° (Figure 3B).

### In Vitro Temperature Monitoring

Heating data was collected for a 2 mm diameter quad‐coil catheter using two MR‐compatible fiber optic temperature probes (FOTEMP‐OEM‐PLUS, Optocon, Dresden, Germany) placed inside the catheter coil and on the coil surface, respectively. The active catheter was suspended within a water bath at room temperature (18 °C). Currents were incremented at 10 mA and steady‐state temperature was recorded after 1 min of coil excitation. Based on these results, maximum temperature changes, Δ*T*_max_, of 5 °C were observed for dual coil excitation at 32 mW. This data indicated similar heating concerns for microcoil excitation as previously studied^[^ ^69^ ^]^ and was used to inform the design process for the QCM.

### Ex Vivo MRI Thermometry

Joule heating safety concerns for the QCM were assessed using noninvasive MRI thermometry. Temperature changes were measured using the change in proton resonant frequency as previously reported.^[^ ^83^ ^]^ Temperature change calibration with a frequency shift using Ringer's solution was first performed for higher accuracy. Later, Ringer's solution was injected inside the renal pelvis to aid catheter steering. Bruker's MAPSHIM method (TR = 22.4 ms, TE1 = 2.4 ms, echo spacing = 7.7 ms, spatial resolution = 1.01 × 1.01 mm^2^, nine slices) based on FASTMAP,^[^ ^84^ ^]^ was used to generate phase images and calculate phase difference in Hz before and after QCM microcoil excitation. Temperature maps were generated consecutively for five different current amplitudes (100, 200, 300, 400, and 500 mA) and later converted to the power intervals shown in Figure 6C. At each interval, microcoils were excited for 1 min with no pause between intervals. Δ*T* values were calculated for nine slices and overlaid onto kidney anatomy scans. Imaging parameters for kidney anatomy scans were as follows: rapid fast spin echo sequence (TR/TEeff 1500/34.085 ms, NEX = 4, RARE factor = 8, spatial resolution = 507 × 507 µm^2^, slice thickness = 1.1 mm, nine slices). Mean Δ*T* values of 4, 6.3, 6.7, 7, and 7.5 °C were observed for 20, 80, 180, 320, and 500 mW, respectively. Figure 6C displays representative temperature heat maps for corresponding power inputs of 20, 180, and 500 mW. This data indicated that power inputs beyond 500 mW were deemed unsafe for use in no flow conditions; this power constraint was used for feasibility studies.

### MR Imaging

The QCM was steered in feasibility experiments including confined ring, kidney, and neurovascular steering under MRI‐guidance. Fast spin echo sequence with the following imaging parameters were used to gather images: ring/kidney steering: (TR/TEeff 500/24.57 ms, RARE factor = 38, 1.01 mm × 1.01 mm spatial resolution, 2 mm slice thickness, one slice, total duration = 500 ms); Neurovascular steering: (TR/TEeff 500/24.57 ms, RARE factor = 38, 1.01 mm × 1.01 mm spatial resolution, 10 mm slice thickness, one slice, total duration = 500 ms).

### Statistical Analysis

Experimental results demonstrated the mean ± standard deviation of the measurements with a sample size (*n*) equal to 3.

## Conflict of Interest

The authors declare no conflict of interest.

## Author Contributions

M.P. conducted the study design, manufacturing, simulations, experiments, data collection, data analysis, and manuscript writing. M.E.T. participated in controller design, simulations, data analysis, and discussions. J.L. participated in the MRI experiments. H.G. participated in discussions in design, manufacturing, and simulations. M.S. participated in discussions, supervision, and manuscript writing.

## Supporting information

Supporting Information

Supplemental Movie 1

Supplemental Movie 2

Supplemental Movie 3

## Data Availability

The data that support the findings of this study are available from the corresponding author upon reasonable request.
