# Supplementary material for: Heat‐Mitigated Design and Lorentz Force‐Based Steering of an MRI‐Driven Microcatheter toward Minimally Invasive Surgery
Source: Adv Sci (Weinh). 2022 Feb 3;9(10):2105352. doi: 10.1002/advs.202105352 (PMC8981448; doi:10.1002/advs.202105352)
Supplement: Supplementary file 1 — Supporting Information [file ADVS-9-2105352-s003.pdf]

ADVANCED SCIENCE
Open Access

Supporting Information

for *Adv. Sci.*, DOI 10.1002/advs.202105352

Heat-Mitigated Design and Lorentz Force-Based Steering of an MRI-Driven Microcatheter toward Minimally Invasive Surgery

*Martin Francis Phelan III, Mehmet Efe Tiryaki, Jelena Lazovic, Hunter Gilbert and Metin Sitti**

ADVANCED SCIENCE

Open Access

Supporting Information

for *Adv. Sci.*, DOI: 10.1002/advs.202105352

Heat-mitigated Design and Lorentz Force-based Steering of an MRI-driven Microcatheter Towards Minimally Invasive Surgery

*Martin Francis Phelan, Mehmet Efe Tiryaki, Jelena Lazovic, Hunter Gilbert Metin Sitti**

Supporting Information

Figure S1. QCM Assembly Exploded View

Figure S2 Heat-mitigated Microcoil Design

Figure S3. Microcoil Design Comparison

Figure S4. Cosserat Model

Figure S5. Conserved Power

Figure S6. MR-compatible Experimental Setup

Figure S7. Embolization Coil Deployment

Table S1. Final Catheter Design Parameters

Movie S1. Endovascular Steering through Narrow Rings

Movie S2. Ex Vivo Kidney Collecting System Navigation for Laser Steering

Movie S3. Neurovascular Navigation for Embolization Coil Deployment

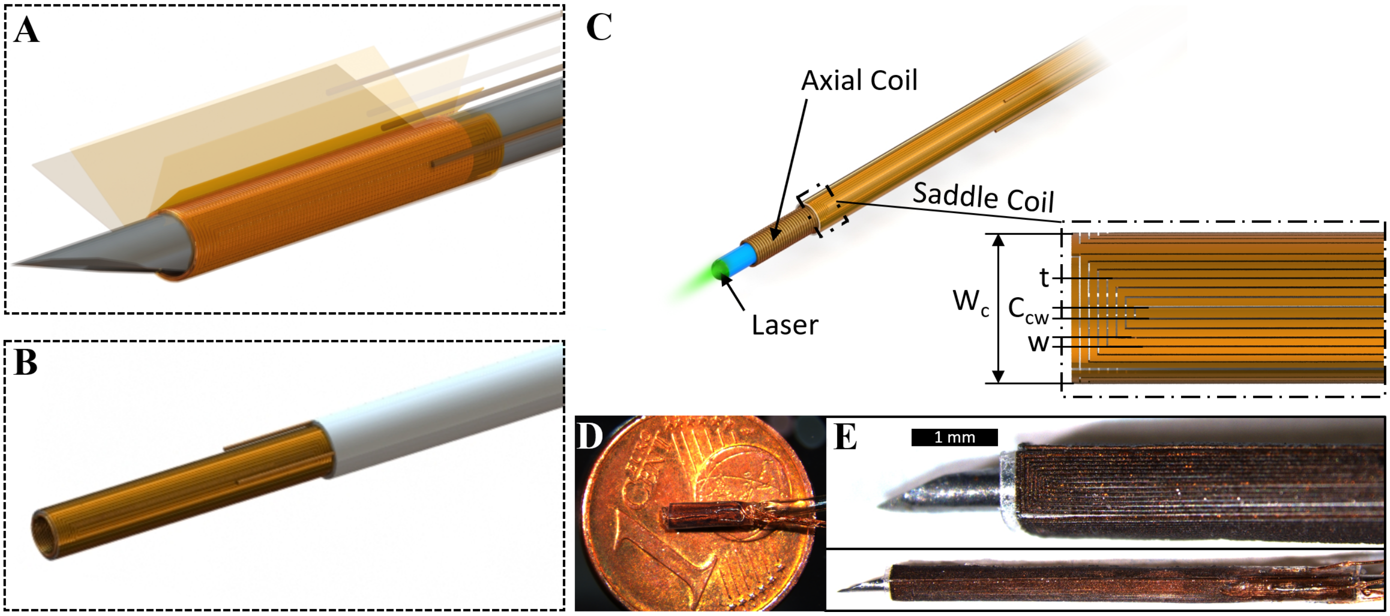

Figure S1: CAD rendering of the saddle coil assembly process and design. **A**) Each quad coil is manually wrapped around the axial coil, the needle is removed, and then attached to the microcatheter **(B)**. **C**) Computer-aided design (CAD) renderings of the active microcatheter with the saddle coil exploded view and coil design parameters. **D**) Photo of 3 mm QCM. **E**) Photo of final saddle coil design and assembled QCM prototype on needle.

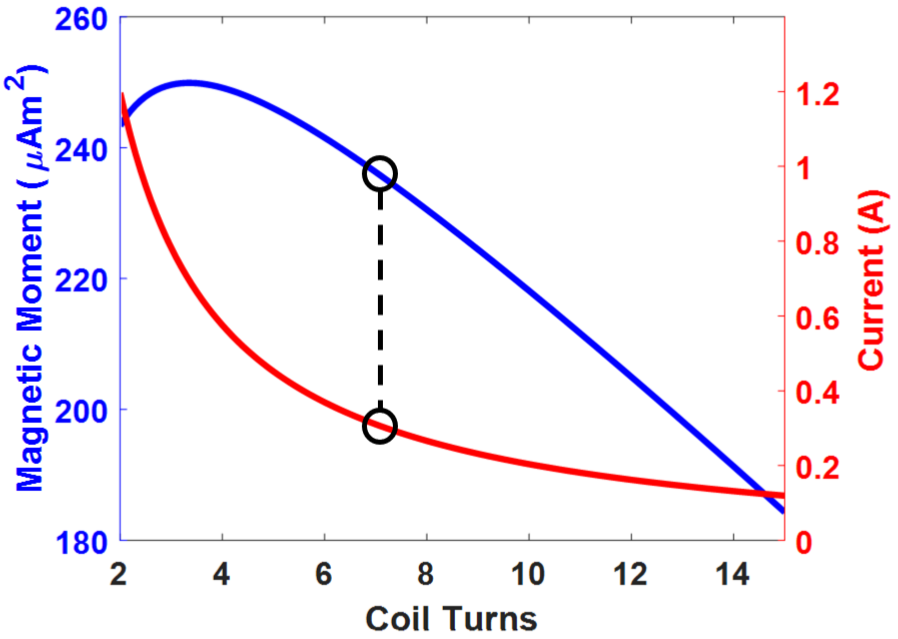

Figure S2: Magnetic moment generated by a single quad-configuration microcoil versus the number of coil turns under constrained power (0.5 W) given an Archimedian spiral coil design. Maximum current to achieve maximum magnetic moment is plotted on the right-side axis.

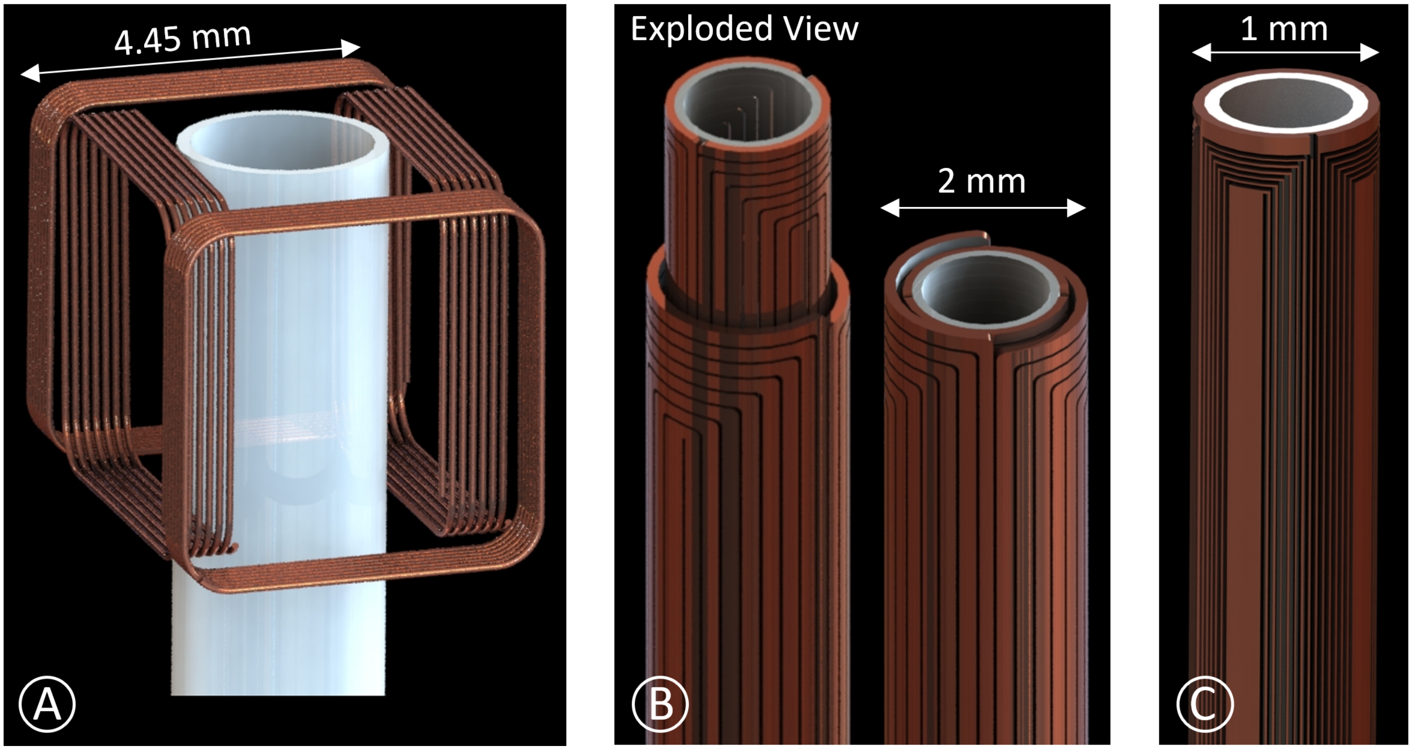

Figure S3: CAD renderings showing the different methods for creating Lorentz force-based catheters (renderings represent visual approximations of other published designs to demonstrate manufacturing approach). A) Each coil is manually wrapped to form an out-of-plane quad-coil configuration [75]. B) Saddle coils are laser-machined on two separate layers and heat shrunk together [65]. C) Proposed quad-coil configuration used in this work in which saddle coils are laser-machined on the same copper sheet leading to smaller, more compact catheter tips.

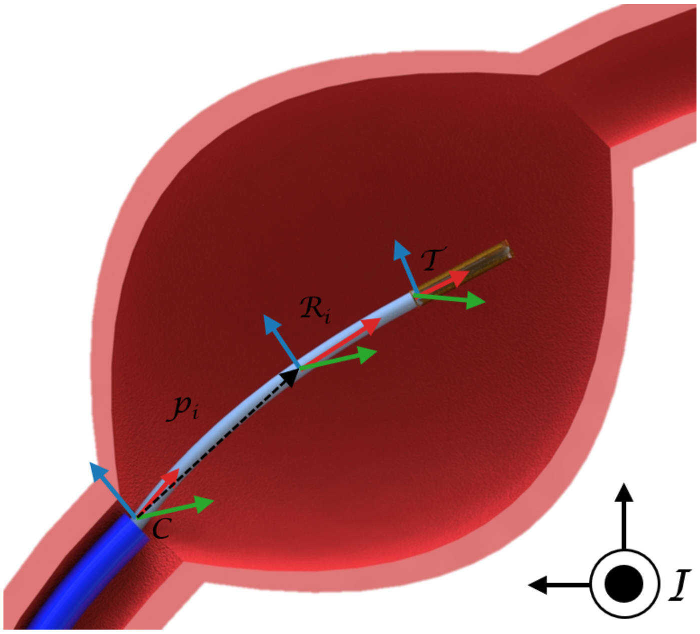

Figure S4: Cosserat rod diagram used to model the nonlinear deformations of the QCM.

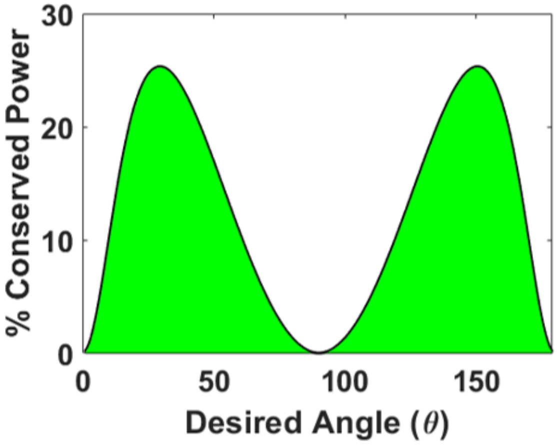

Figure S5: Conserved power using power-optimized controller for both initial orientation cases ($\gamma = 0°$) and ($\gamma = 90°$).

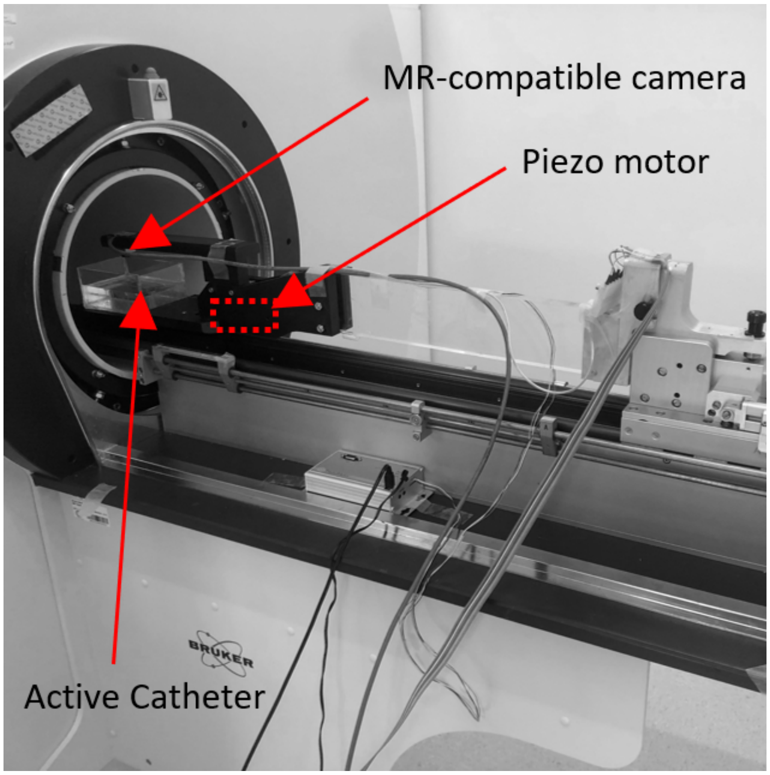

Figure S6: Photo of the MR-compatible setup using 3D-printed components on the MR scanner linear stage.

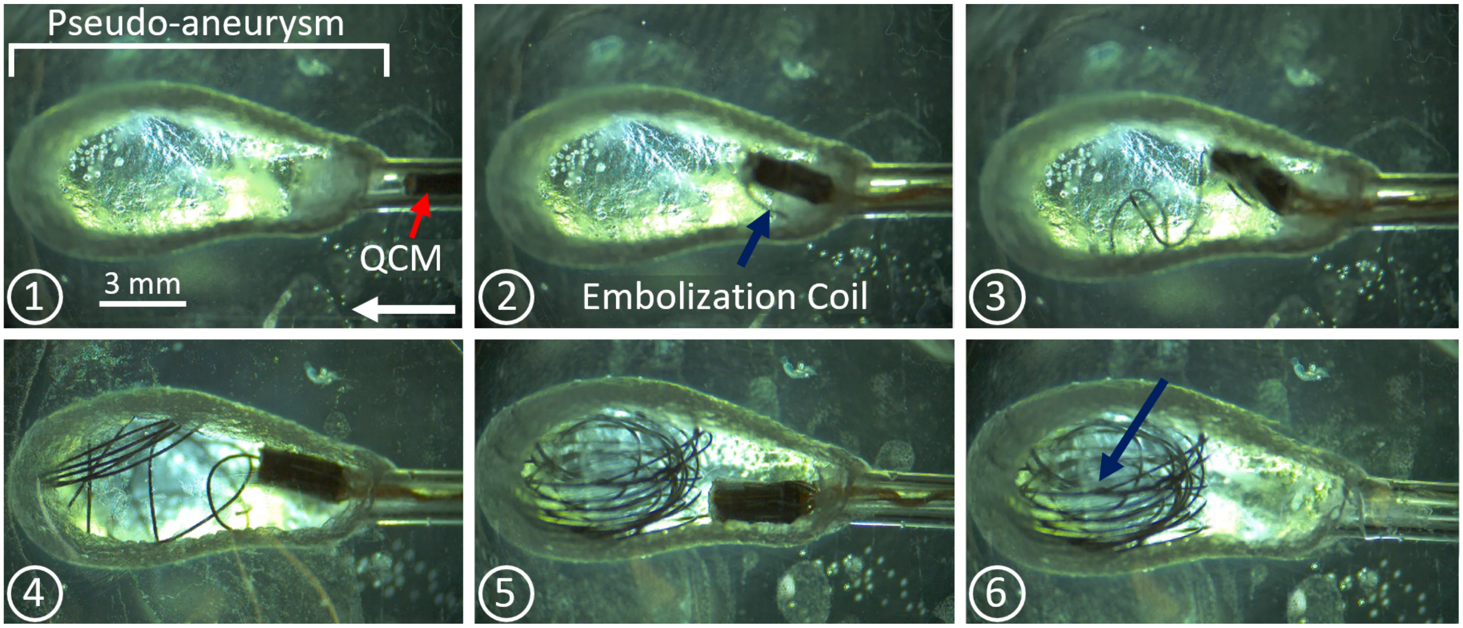

Figure S7: Photos of the embolization coil deployment process using a pseudo-aneurysm polyurethane model. 1) The QCM, indicated by the red arrow, advances into the aneurysm and 2) begins deploying the coil, shown by the blue arrow. 3-5) The aneurysm is filled by the embolization coil and 6) the QCM is retracted.

Table S1: Final catheter design parameters ($\theta_{des} = 180°$)

Parameter	Description	Value
W_c	Saddle coil width	0.76 [mm]
D_c	Catheter diameter	0.96 [mm]
t	Gap spacing	10 [μm]
C_{cw}	Coil core width	0.05 [mm]
s	Wire thickness	18 [μm]
w	Wire width	40 [μm]
N_{saddle}	Saddle coil turns	7
E	Catheter Young's modulus	24.13 [MPa]
pw_D	Power wire diameter	80 [μm]
L	Catheter insertion length	60 [mm]
L_c	Coil length	9.54 [mm]
P_{total}	Total power	0.44 [W]
N_{axial}	Axial coil turns	250
I_{saddle}	Saddle coil current	-259 [mA]
I_{axial}	Axial coil current	0 [mA]
